# Clinical Significance and Prognostic Value of the Expression of LAMP3 in Oral Squamous Cell Carcinoma

**DOI:** 10.1155/2017/1218254

**Published:** 2017-05-14

**Authors:** Jun Lu, Hengcheng Ma, Shuijin Lian, Dan Huang, Min Lian, Ye Zhang, Jianfei Huang, Xingmei Feng

**Affiliations:** ^1^Department of Stomatology, Affiliated Hospital of Nantong University, Nantong, Jiangsu, China; ^2^Department of Pathology, Affiliated Hospital of Nantong University, Nantong, Jiangsu, China

## Abstract

Recent studies demonstrated high expression of lysosome-associated membrane protein 3 (LAMP3) in a variety of malignancies including esophageal squamous cell carcinoma, gastrointestinal cancer, breast cancer, and cervical cancer and its involvement in several biological activities of tumor cells. However, the expression of LAMP3 and its value in oral squamous cell carcinoma (OSCC) remain unclear. In this study, we examined the expression of LAMP3 in OSCC tissue samples and investigated the relationship between LAMP3 and clinical characteristics of patients with OSCC. We examined mRNA and protein levels of LAMP3 in OSCC tissues and neighboring normal tissues using quantitative real-time polymerase chain reaction and immunohistochemistry analyses, respectively. Both the mRNA and protein levels of LAMP3 were significantly higher in OSCC tissues than in adjacent normal tissues. Chi-square analysis showed that the high LAMP3 expression was notably linked to the degree of tumor differentiation and advanced TNM stage. Univariate and multivariate analyses showed that the high LAMP3 expression was an independent prognostic marker in OSCC. Our results suggest that LAMP3 might act as a potential anticancer target and a prognostic marker in patients with OSCC.

## 1. Introduction

Oral squamous cell carcinoma (OSCC) constitutes a large subgroup of head and neck squamous cell carcinoma and occupies more than 90% of malignancies in the oral cavity [1]. OSCC is a tobacco- and alcohol-related cancer; however, it also can develop in the absence of tobacco and alcohol consumption [2]. More than 300,000 new cases of OSCC are diagnosed annually worldwide [3], and the incidence rate of OSCC is continuously increasing in many countries [4].

The conventional treatment strategy for OSCC includes surgery, radiation therapy, or both surgery and radiation therapy. Treatment methods of advanced OSCC include surgical resection with postoperative adjuvant radiotherapy. A primary aim of oncologic surgery is to achieve curative resection with histological tumor-free margins [5]. Adequate surgical resection is crucial for local control and personalized postoperational management [6–8]. This disease frequently appears along with metastasis, high recurrence, and poor prognosis due to late detection or diagnosis at advanced stages [9]. Despite improvement in early diagnosis and progress in therapy, the outcomes of the disease have remained high, with 30% local or regional recurrence and 25% distant metastasis, leading to an unfavorable 5-year survival rate (about 50%) [10]. Therefore, exploring new methods of diagnosis and seeking novel molecular markers that can predict the prognosis of patients with OSCC for management of OSCC are urgently needed.

Lysosome-associated membrane protein 3 (LAMP3) belongs to the LAMP protein family, which was initially indicated as a molecular marker of mature dendritic cells (CD208, DC-LAMP) [11]. Recent studies have demonstrated that increased expression of LAMP3 correlated with unfavorable prognosis of patients with esophageal squamous cell carcinoma [12], gastrointestinal stromal tumor (GIST) [13], breast cancers [14, 15], cervical cancer [16], and head and neck squamous cell carcinomas [17]. LAMP3 is also reported to induce and promote migration and invasion of tumor cells [14, 18, 19]. Moreover, a positive significant relationship between the expression of LAMP3 and lymph node metastasis has been reported [13, 16, 20]. Thus, LAMP3 might serve as a potential molecular marker for the survival of cancer patients. To date, however, the expression of LAMP3 in OSCC patients and the relationship between LAMP3 and clinical characteristics of these patients have not been examined.

Here, we determined the mRNA and protein expression of LAMP3 in OSCC tissue samples and neighboring normal tissues using quantitative real-time polymerase chain reaction (qPCR) and immunohistochemistry (IHC) analyses, respectively. Moreover, the relationship between LAMP3 and clinical characteristics of OSCC patients was also analyzed. Our findings suggested that the expression of LAMP3 might serve as a new indicator of unfavorable survival and provide evidence that LAMP3 could be an innovative anticancer target for OSCC therapy.

## 2. Material and Methods

### 2.1. Tissue Samples and Patient Characteristics

A total of 248 OSCC tissue samples (including 107 buccal squamous cell carcinoma (BSCC) and 141 tongue squamous cell carcinoma (TSCC)) and 55 control samples (including 32 normal oral mucosas and 23 chronic inflammations) were collected for IHC analysis. Additional 25 frozen OSCC tissues and 25 normal oral tissues as controls were collected for mRNA determination using qPCR. All clinical characteristics, such as gender, age, habits (including tobacco and alcohol consumption), differentiation, tumor location, and stages of T, N, and TNM, were obtained from the medical records of patients in Affiliated Hospital of Nantong University, Nantong, Jiangsu, China. All the patients did not receive preoperative radiotherapy, immunotherapy, or chemotherapy. The research protocol was authorized by the Human Research Ethics Committee of the local hospital.

### 2.2. qRT-PCR

Total RNA was isolated from fresh frozen-tissues using the RNeasy Plus Mini Kit (Qiagen, Hilden, Germany) and converted to cDNA using a High Capacity RNA-to-cDNA Kit (Life Technologies, Carlsbad, CA, USA). Next, qRT-PCR was performed using the Power SYBR Green PCR Master Mix (Life Technologies, Carlsbad, CA, USA) following the manufacturer's instructions. The primers for LAMP3 were as follows: forward primer (5′-CCTTCAAGTGCGTGAGTGAA-3′) and reverse primer (5′-CCATAAGGCAGAGACCAACC-3′). The primers for *β*-actin were as follows: forward primer (5′-TTAATCTTCGCCTTAATACTT-3′) and reverse primer (5′-AGCCTTCATACATCTCAA-3′). The relative expression level of LAMP3 was calculated using the ΔΔCt method. Amplification conditions consisted of the following steps: 10 min at 95°C for Taq activation followed by 40 cycles of 95°C for 15 s and 60°C for 1 min. All experiments were performed in triplicate.

### 2.3. IHC Staining

We used IHC staining to determine the protein expression of LAMP3 in 303 documented paraffin-fixed tissue specimens (including 107 BSCC, 141 TSCC, and 55 matched noncancerous oral tissues). The whole tissue blocks were constructed to tissue microarrays (TMA) in preparation. TMAs were then deparaffinized with 100% xylene and rehydrated in a graded alcohol. Antigen retrieval was performed by boiling in citrate buffer (pH 6.0) for 10 min in an autoclave, followed by quenching in 3% hydrogen peroxide to block endogenous peroxidase activity. After washing with phosphate-buffered saline (PBS), slides were incubated with rabbit polyclonal anti-LAMP3 antibody (ab111090; 1 : 100 dilution; Abcam, Cambridge, MA, USA) at 4°C overnight. On the next day, sections were incubated with biotinylated goat anti-rabbit secondary antibody (SN135; 1 : 1000 dilution; Beyotime Institute of Biotechnology, Haimen, China) at room temperature for 30 min. Following washing with PBS, the slides were processed using horseradish peroxidase and dyed with 3,3-diaminobenzidine (DAB) chromogen solution. Finally, the sections were counterstained with hematoxylin, dehydrated, and coverslipped.

Two independent pathologists evaluated the results of IHC staining in a double-blind manner. The expression of LAMP3 was scored as described in a previous study [21]. Briefly, we scored the percentage of LAMP3-positive cells as follows: 0 for 0% staining, 1 for 1%–33%, 2 for 34%–66%, and 3 for 67%–100%. The intensity of LAMP3 staining was scored as follows: 0 for no staining, 1 for weak staining, 2 for moderate staining, and 3 for strong staining. The product of the percentage and intensity scores was taken as the final staining score (range from 0% to 300%) [22]. The X-tile software program (The Rimm Lab, Yale University; http://www.tissuearray.org/rimmlab) was used to determine the cutoff point for the IHC staining score of the expression of LAMP3 to analyze the overall survival [21]. We defined 140% as the cutoff point in LAMP3 level in cancer cells (*P* = 0.013). Samples higher than 140% were categorized as high expression, and samples lower than 140% were categorized as low expression.

### 2.4. Statistical Analysis

The SPSS20.0 software (SPSS, Inc., Chicago, IL, USA) was used for statistical analysis as described previously [21]. A nonparametric test, Manny-Whitney *U* test, was performed for comparing the mRNA expression of LAMP3 between OSCC tissues and normal tissues. Chi-square analysis was used to compare the differences between the expression of LAMP3 and clinical characteristics of patients with OSCC. The Cox proportional hazards model was used to calculate the univariate and multivariate analyses. Kaplan-Meier method was used to draw survival curves. *P* value <0.05 was deemed to be statistically significant for all analyses.

## 3. Results

### 3.1. The mRNA Level of LAMP3 Was Increased in OSCC Tissues Compared with That in Adjacent Normal Samples

**Figure 1:**
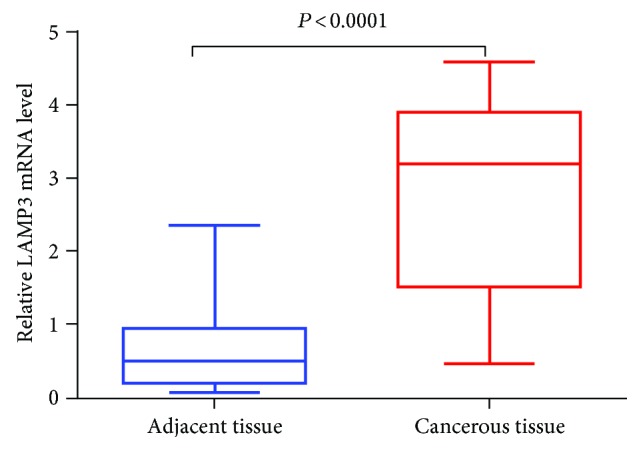
The mRNA expression of LAMP3 in OSCC and tumor-adjacent tissues was determined by qPCR. The mRNA expression of LAMP3 (normalized to that of *β*-actin) was significantly higher in cancerous tissues compared with adjacent normal tissues by Mann-Whitney *U* test (*P* < 0.0001).

We performed qPCR to measure expression level of LAMP3 mRNA in OSCC tissues and normalized LAMP3 mRNA levels to mRNA levels of the housekeeping gene *β*-actin [23, 24]. LAMP3 mRNA levels were significantly elevated in cancerous tissues compared with adjacent normal control samples by the Mann-Whitney *U* test (*P* < 0.0001) (Figure 1).

### 3.2. LAMP3 Protein Level Was Significantly Increased in OSCC Tissues Compared with That in Adjacent Normal Tissues

**Figure 2:**
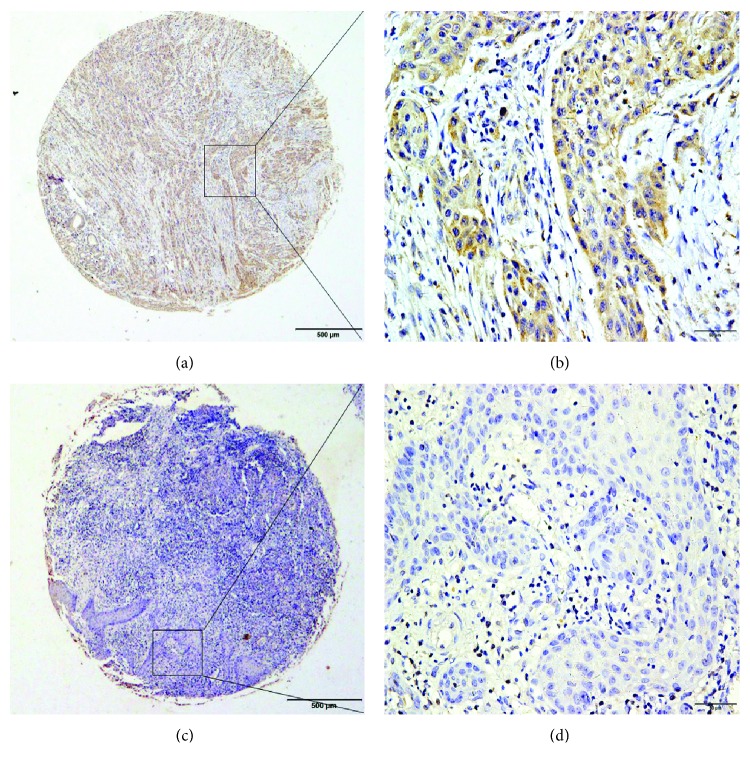
The protein expression of LAMP3 in OSCC and normal tissues was determined by IHC analysis in tissue microarray. (a, b) Strong positive cytoplasmic expression of LAMP3 in OSCC. (c, d) Negative IHC staining of LAMP3 in a normal tissue sample. (a, c) Original magnification ×40. (b, d) Original magnification ×400.

IHC staining showed that LAMP3 was detectable primarily in the cytoplasm of cancer cells and presented as brown particles (Figure 2), consistent with previous studies [25]. The frequency of high LAMP3 protein expression in the cytoplasm of cancer cells was significantly higher in OSCC tissues than in the normal oral mucosa (*P* < 0.001) (Table 1). Low or no positive staining was found in the nuclei of cancer cells or stromal cells, while no positive signals were found in the normal oral mucosa. We classified samples based on high or low LAMP3 expression, as described in Methods. A total of 62 (25%) cases were categorized as high LAMP3 expression, and 186 (75%) cases were categorized as low LAMP3 expression.

### 3.3. Relationship Between the Expression of LAMP3 And Clinical Characteristics of OSCC Patients

We next examined the correlation of LAMP3 expression with clinical features of patients with OSCC. We found that high protein level of LAMP3 was significantly linked to the degree of differentiation (*P* = 0.011) and advanced TNM stage (*P* = 0.043) (Table 2).

### 3.4. High Expression of LAMP3 Predicts Poor Overall Survival in Patients with OSCC

Finally, the prognostic factors were analyzed in patients with OSCC using both univariate and multivariate analyses. In univariate analysis, high expression of LAMP3 (*P* = 0.013), low degree of tumor differentiation (*P* = 0.018), node metastasis (*P* < 0.001), and advanced TNM stage (*P* = 0.001) were markedly related to the overall survival in patients with OSCC. The T stage was marginally associated with harmful prognosis (*P* = 0.056). In the multivariate analysis, high expression of LAMP3 (*P* = 0.048) was significantly linked to the poor overall survival, similarly as the degree of tumor differentiation (*P* = 0.020) and advanced TNM stage (*P* = 0.001) were always regarded as significant predictors of poor prognosis of OSCC patients (Table 3).

**Figure 3:**
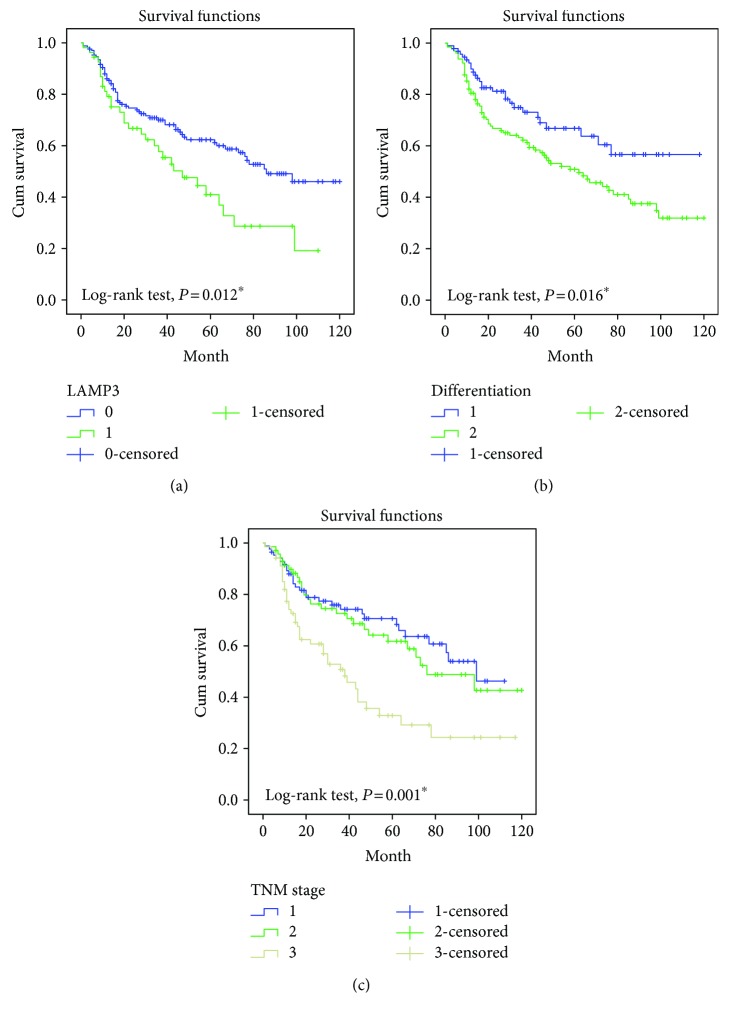
Kaplan-Meier survival analysis was performed in patients with OSCC. (a) Overall survival rate in patients with high cytoplasmic expression of LAMP3 (green line) was significantly lower than that in patients with no or low cytoplasmic expression of LAMP3 (blue line). (b) Overall survival rate in patients with poorly differentiated tumors (green line) was significantly lower than that in patients with moderately to highly differentiated tumors (blue line). (c) Overall survival rate in patients with advanced TNM stage (stage IV, yellow line) was significantly lower than that in patients with early TNM stage (stages II-III, green line; stages 0-I, blue line).

Kaplan-Meier survival curves showed that OSCC patients with high expression of LAMP3 exhibited a significantly poor survival time compared with those with low expression of LAMP3 (Figure 3(a)). Moreover, low degree of differentiation was associated with an unfavorable survival time (Figure 3(b)). The overall survival rate of patients with an advanced stage TNM (stage IV (yellow line)) was lower than that of patients with early-stage disease (stages 0-I (blue line) and disease at stages II-III (green line)) (Figure 3(c)).

## 4. Discussion

With increasing incidence and mortality, cancer is the leading cause of death in China and is a major public health problem. Because of the massive population in China, which constitutes approximately one-fifth of the world population, cancer cases in China significantly contribute to the global burden of cancer: almost 22% of new global cancer cases and close to 27% of global cancer deaths occur in China [26]. The incidence of OSCC also continues to grow. OSCC is a highly invasive and metastatic malignancy, and despite advances in surgery, radiation, and chemotherapy, the 5-year disease-free survival (DFS) is about 50% [27], with frequent local recurrence and metastasis. To date, studies seeking potential predictive molecular markers for OSCC have been extensively performed and some markers were identified as significantly correlating with tumor biological response and clinical features in vitro and in vivo [28–32]. Some molecular markers are even enable to significantly influence survival [32, 33].

Previous studies reported that LAMPs are involved in human malignancies. Carlsson et al. report that LAMPs can relocalize to the plasma membrane in several types of cancer cells [34]. Saitoh et al. found that the elevated expression of LAMP1 and LAMP2 predicts high metastatic viability in colon cancer cell lines [35]. These observations indicate that LAMPs might promote the metastasis of malignant tumors.

LAMP3 was first identified as a lung-specific gene [28] and now represents a well-established cell surface marker of mature dendritic cells. Overexpression of LAMP3 has been found in several human cancers such as lung, colon, esophagus, breast, and ovary cancers [36] and correlated with node metastasis by affecting cell migration [16, 19]. Dominguez-Bautista et al. demonstrated that LAMP3 is required for cell survival during proteasomal inhibition in vitro [37]. In addition, the protein expression of LAMP3 contributes to locoregional recurrence in breast cancer [15, 18]. Nagelkerke et al. reported that LAMP3 is also involved in tamoxifen resistance in MCF7 breast cancer cells through the modulation of autophagy, while knockdown of this gene presents an increased sensitivity toward tamoxifen [38]. In addition, high LAMP3 expression predicts poor survival in patients with cervix cancer and esophageal squamous cell carcinoma [12, 16]. Moreover, LAMP3 combined with TP53 determination can help predict poor outcome in patients with laryngeal squamous cell carcinoma (LSCC) and GIST [13, 17]. However, the expression of LAMP3 and its relationship with the clinical features of OSCC patients have remained unclear. Here, we investigated the potential role of LAMP3 in OSCC development.

We first examined LAMP3 mRNA expression in OSCC tissues and surrounding normal oral tissues using qPCR and found a significantly higher level of LAMP3 mRNA in malignant tissues. Consistent with the qPCR result, IHC staining on 248 paraffin-embedded OSCC and 55 matched noncancerous specimens showed that LAMP3 protein expression was higher in OSCC compared with normal tissues. Chi-square analysis revealed a significantly positive correlation of high expression of LAMP3 with a low degree of tumor differentiation (*P* = 0.011) and an advanced TNM stage (*P* = 0.043). Univariate and multivariate analyses exhibited that positive LAMP3 expression, degree of differentiation, and TNM stage were independent prognostic factors affecting the survival of patients with OSCC, and Kaplan-Meier analysis showed that the lifespan of patients with positive LAMP3 expression was shorter than that of patients with negative expression.

More than half of OSCCs evolve from oral precancerous lesions (OPLs) in the oral cavity. Thus, early diagnosis and intervention of OSCCs can increasingly prolong the survival of patients and improve their quality of life. Furthermore, the recognition of squamous cell hyperplasia or dysplasia in the oral cavity might be helpful in ameliorating strategies during the process of oral oncogenesis at the precancerous stage; however, the relevant knowledge is lacking [39–41]. In this study, the expression of LAMP3 was compared between cancerous tissues of OSCC and normal oral mucosa, and we found that LAMP3 was highly expressed in tumor cells. Furthermore, high expression of LAMP3 was linked to poor prognosis. Future research with a large number of OPL cases is urgently needed to determine the expression of LAMP3 and analyze the differences between OPL and OSCC.

## 5. Conclusion

This study investigated the potential role of LAMP3 in OSCC. The expression of LAMP3 was determined in cancerous OSCC tissues and matched normal tissues using qPCR and IHC methods, respectively. A comparison between the expression of LAMP3 and clinical characteristics of patients with OSCC was performed, and the survival curves were drawn. In the current study, we found that higher expression of LAMP3 in OSCC tissues than in control samples may be a novel prognostic marker and a potential anticancer target for OSCC patients.

## Figures and Tables

**Table 1:** LAMP3 expression in cancerous and noncancerous oral tissues.

Characteristics	*n*	Low LAMP3 expression (%)	High LAMP3 expression (%)	Pearson *χ*2	*P*
OSCC	248	186 (75.00)	62 (25.00)	12.332	<0.001^∗^
Noncancerous oral tissues	55	53 (96.36)	2 (3.64)		

^∗^ *P* < 0.05.

**Table 2:** Relationship between the expression of LAMP3 and clinicopathological characteristics in OSCC.

Characteristic		*n*	Low expression (%)	High expression (%)	Pearson *χ*2	*P*
Total		248	186 (75.00)	62 (25.00)		
Gender	Male	115	88 (76.52)	27 (23.48)	0.265	0.607
	Female	133	98 (73.68)	35 (26.32)		
Age	<60	110	79 (71.82)	31 (28.18)	1.067	0.302
	≥60	138	107 (77.54)	31 (22.46)		
Tobacco consumption	No	134	98 (73.13)	36 (26.87)	0.541	0.462
	Yes	114	88 (77.19)	26 (22.81)		
Alcohol consumption	No	132	97 (73.48)	35 (26.52)	0.346	0.557
	Yes	116	89 (76.72)	27 (23.38)		
Tumor location	Buccal	107	77 (71.96)	30 (28.04)	0.926	0.336
	Tongue	141	109 (77.30)	32 (22.70)		
Differentiation	Poor	102	85 (83.33)	17 (16.67)	6.417	0.011^∗^
	Moderate and well	146	101 (69.18)	45 (30.28)		
TNM stage	0-I	95	76 (80.00)	19 (20.00)	6.305	0.043^∗^
	II-III	73	58 (79.45)	15 (20.55)		
	IV	80	52 (65.00)	28 (35.00)		
T	Tis-T1	110	82 (74.55)	28 (25.45)	0.486	0.784
	T2	58	42 (72.41)	16 (27.59)		
	T3-T4	80	62 (77.50)	18 (22.50)		
Node metastasis	No	193	150 (77.72)	43 (22.28)	3.434	0.064
	Yes	55	36 (65.45)	19 (34.55)		

^∗^ *P* < 0.05.

**Table 3:** Univariate and multivariate analyses of prognostic factors in OSCC for 5-year survival.

Variable	Univariate analysis			Multivariate analysis		
	HR	*P* value	95% CI	HR	*P* value	95% CI
LAMP3 expression	1.722	0.013^∗^	1.119–2.648	1.546	0.048^∗^	1.003–2.383
High versus low						
Gender	1.040	0.847	0.696–1.556			
Female versus male						
Age (years)	1.259	0.273	0.834–1.898			
<60 versus ≥60						
Tobacco consumption	0.847	0.420	0.565–1.268			
Yes versus no						
Alcohol consumption	0.889	0.569	0.592–1.334			
Yes versus no						
Tumor location	0.983	0.934	0.655–1.474			
Buccal versus tongue						
Differentiation	1.715	0.018^∗^	1.097–2.682	1.707	0.020^∗^	1.089–2.674
Well versus moderate versus poor						
T stage	1.257	0.056	0.994–1.589			
Tis-1 versus T2 versus T3-4						
Node metastasis	3.009	<0.001^∗^	1.976–4.582			
No versus yes						
TNM stage	1.562	0.001^∗^	1.213–2.011	1.566	0.001^∗^	1.213–2.022
Stages 0-I versus stages II-III versus stage IV						

^∗^ *P* < 0.05.

## References

[1] Perez-Sayans M., Somoza-Martin J. M., Barros-Angueira F., Reboiras-López M. D., Gándara Rey J. M., García-García A. (2009). Genetic and molecular alterations associated with oral squamous cell cancer (review). *Oncology Reports*.

[2] Markopoulos A. K. (2012). Current aspects on oral squamous cell carcinoma. *The Open Dentistry Journal*.

[3] Torre L. A., Bray F., Siegel R. L., Ferlay J., Lortet-Tieulent J., Jemal A. (2015). Global cancer statistics, 2012. *CA: A Cancer Journal for Clinicians*.

[4] Simard E. P., Torre L. A., Jemal A. (2014). International trends in head and neck cancer incidence rates: differences by country, sex and anatomic site. *Oral Oncology*.

[5] Pfister D. G., Spencer S., Brizel D. M. (2015). Head and neck cancers, version 1.2015. *Journal of the National Comprehensive Cancer Network*.

[6] Jerjes W., Upile T., Petrie A. (2010). Clinicopathological parameters, recurrence, locoregional and distant metastasis in 115 T1-T2 oral squamous cell carcinoma patients. *Head & Neck Oncology*.

[7] Al-Rajhi N., Khafaga Y., El-Husseiny J. (2000). Early stage carcinoma of oral tongue: prognostic factors for local control and survival. *Oral Oncology*.

[8] Slootweg P. J., Hordijk G. J., Schade Y., van Es R. J., Koole R. (2002). Treatment failure and margin status in head and neck cancer. A critical view on the potential value of molecular pathology. *Oral Oncology*.

[9] Warnakulasuriya S. (2009). Global epidemiology of oral and oropharyngeal cancer. *Oral Oncology*.

[10] Zhang H., Dziegielewski P. T., Biron V. L. (2013). Survival outcomes of patients with advanced oral cavity squamous cell carcinoma treated with multimodal therapy: a multi-institutional analysis. *Journal of Otolaryngology - Head & Neck Surgery*.

[11] de Saint-Vis B., Vincent J., Vandenabeele S. (1998). A novel lysosome-associated membrane glycoprotein, DC-LAMP, induced upon DC maturation, is transiently expressed in MHC class II compartment. *Immunity*.

[12] Liao X., Chen Y., Liu D., Li F., Li X., Jia W. (2015). High expression of LAMP3 is a novel biomarker of poor prognosis in patients with esophageal squamous cell carcinoma. *International Journal of Molecular Sciences*.

[13] Sun R., Wang X., Zhu H. (2014). Prognostic value of LAMP3 and TP53 overexpression in benign and malignant gastrointestinal tissues. *Oncotarget*.

[14] Nagelkerke A., Bussink J., Mujcic H. (2013). Hypoxia stimulates migration of breast cancer cells via the PERK/ATF4/LAMP3-arm of the unfolded protein response. *Breast Cancer Research*.

[15] Nagelkerke A., Mujcic H., Bussink J. (2011). Hypoxic regulation and prognostic value of LAMP3 expression in breast cancer. *Cancer*.

[16] Kanao H., Enomoto T., Kimura T. (2005). Overexpression of LAMP3/TSC403/DC-LAMP promotes metastasis in uterine cervical cancer. *Cancer Research*.

[17] Qiu X., You Y., Huang J., Wang X., Zhu H., Wang Z. (2015). LAMP3 and TP53 overexpression predicts poor outcome in laryngeal squamous cell carcinoma. *International Journal of Clinical and Experimental Pathology*.

[18] Mujcic H., Rzymski T., Rouschop K. M. (2009). Hypoxic activation of the unfolded protein response (UPR) induces expression of the metastasis-associated gene LAMP3. *Radiotherapy and Oncology*.

[19] Mujcic H., Nagelkerke A., Rouschop K. M. (2013). Hypoxic activation of the PERK/eIF2α arm of the unfolded protein response promotes metastasis through induction of LAMP3. *Clinical Cancer Research*.

[20] Zhai X., Zhu H., Wang W., Zhang S., Zhang Y., Mao G. (2014). Abnormal expression of EMT-related proteins, S100A4, vimentin and E-cadherin, is correlated with clinicopathological features and prognosis in HCC. *Medical Oncology*.

[21] Lian S., Zhai X., Wang X. (2016). Elevated expression of growth-regulated oncogene-alpha in tumor and stromal cells predicts unfavorable prognosis in pancreatic cancer. *Medicine (Baltimore)*.

[22] Han L., Tang M. M., Xu X. (2016). LTBP2 is a prognostic marker in head and neck squamous cell carcinoma. *Oncotarget*.

[23] Harada K., Kawashima Y., Yoshida H., Sato M. (2006). Thymidylate synthase expression in oral squamous cell carcinoma predicts response to S-1. *Oncology Reports*.

[24] Kalinke L. P., Alvares L. E., Schussel J. L. (2016). Expression of WNT10A gene in oral squamous cell carcinoma. *The West Indian Medical Journal*.

[25] Treilleux I., Blay J. Y., Bendriss-Vermare N. (2004). Dendritic cell infiltration and prognosis of early stage breast cancer. *Clinical Cancer Research*.

[26] Ferlay J., Soerjomataram I., Dikshit R. (2015). Cancer incidence and mortality worldwide: sources, methods and major patterns in GLOBOCAN 2012. *International Journal of Cancer*.

[27] Kang H., Kiess A., Chung C. H. (2015). Emerging biomarkers in head and neck cancer in the era of genomics. *Nature Reviews. Clinical Oncology*.

[28] Lee J. C., Chiang K. C., Feng T. H. (2016). The iron chelator, Dp44mT, effectively inhibits human oral squamous cell carcinoma cell growth in vitro and in vivo. *International Journal of Molecular Sciences*.

[29] Hwang-Bo J., Park J. H., Bae M. G., Chung I. S. (2016). Recombinant canstatin inhibits VEGF-A-induced lymphangiogenesis and metastasis in an oral squamous cell carcinoma SCC-VII animal model. *Cancer Medicine*.

[30] Chen C., Shin J. H., Eggold J. T. (2016). ESM1 mediates NGFR-induced invasion and metastasis in murine oral squamous cell carcinoma. *Oncotarget*.

[31] Straub M., Drecoll E., Pfarr N. (2016). CD274/PD-L1 gene amplification and PD-L1 protein expression are common events in squamous cell carcinoma of the oral cavity. *Oncotarget*.

[32] Kumar M., Mehra S., Thakar A. (2016). End binding 1 (EB1) overexpression in oral lesions and cancer: a biomarker of tumor progression and poor prognosis. *Clinica Chimica Acta*.

[33] Saghravanian N., Zamanzadeh M., Meshkat Z., Afzal Aghaee M., Salek R. (2016). Evaluation of the prevalence rate and the prognostic effect of human papilloma virus infection in a group of patients with oral cavity squamous cell carcinoma. *Iran Journal of Cancer Prevention*.

[34] Carlsson S. R., Fukuda M. (1992). The lysosomal membrane glycoprotein lamp-1 is transported to lysosomes by two alternative pathways. *Archives of Biochemistry and Biophysics*.

[35] Saitoh O., Wang W. C., Lotan R., Fukuda M. (1992). Differential glycosylation and cell surface expression of lysosomal membrane glycoproteins in sublines of a human colon cancer exhibiting distinct metastatic potentials. *The Journal of Biological Chemistry*.

[36] Ozaki K., Nagata M., Suzuki M. (1998). Isolation and characterization of a novel human lung-specific gene homologous to lysosomal membrane glycoproteins 1 and 2: significantly increased expression in cancers of various tissues. *Cancer Research*.

[37] Dominguez-Bautista J. A., Klinkenberg M., Brehm N. (2015). Loss of lysosome-associated membrane protein 3 (LAMP3) enhances cellular vulnerability against proteasomal inhibition. *European Journal of Cell Biology*.

[38] Nagelkerke A., Sieuwerts A. M., Bussink J. (2014). LAMP3 is involved in tamoxifen resistance in breast cancer cells through the modulation of autophagy. *Endocrine-Related Cancer*.

[39] Matta A., Ralhan R., DeSouza L. V., Siu K. W. (2010). Mass spectrometry-based clinical proteomics: head-and-neck cancer biomarkers and drug-targets discovery. *Mass Spectrometry Reviews*.

[40] Kaur J., Sawhney M., DattaGupta S. (2013). Clinical significance of altered expression of β-catenin and E-cadherin in oral dysplasia and cancer: potential link with ALCAM expression. *PloS One*.

[41] Siegel R., Ma J., Zou Z., Jemal A. (2014). Cancer statistics, 2014. *CA: A Cancer Journal for Clinicians*.

